# Taxonomic revision of the *Ophiocordyceps ravenelii* complex (Hypocreales, Ophiocordycipitaceae) from China, with an emended description and a new species

**DOI:** 10.3897/mycokeys.133.192411

**Published:** 2026-06-08

**Authors:** Quan-Ying Dong, Nian-Kai Zeng, Chao Hu, Chang-Kun Liu, Jin-Na Zhou, Jin-Lin Liu, Shun-Yu Gao, Cheng-Dong Xu

**Affiliations:** 1 College of Agronomy, Chuxiong Normal University, Chuxiong 675000, China Chuxiong Normal University Chuxiong China; 2 Ministry of Education Key Laboratory for Ecology of Tropical Islands, Key Laboratory of Tropical Animal and Plant Ecology of Hainan Province, College of Life Sciences, Hainan Normal University, Haikou 571158, China Hainan Normal University Haikou China; 3 Key Laboratory of East China Plant Conservation and Utilization, National Forestry and Grassland Administration, Shanghai Chenshan Botanical Garden, Shanghai, 201602, China Shanghai Chenshan Botanical Garden Shanghai China

**Keywords:** Entomopathogenic fungi, new taxon, phylogeny, species diversity, taxonomy

## Abstract

The *Ophiocordyceps ravenelii* complex (Ophiocordycipitaceae, Hypocreales) predominantly parasitizes Coleopteran larvae, yet its species diversity remains poorly documented, particularly on non-scarabaeid hosts. Based on morphological comparisons and multi-locus phylogenetic analyses (nrSSU, nrLSU, *tef1-α*, *rpb1*, *rpb2*), we present a taxonomic revision of this complex from Yunnan Province, southwestern China. Specimens CXAC 0024 and CXAC 0025 are identified as *Ophiocordyceps jinguangensis*, for which an emended description is provided, documenting its occurrence on Elateroidea larvae and expanding its known morphological variability. *Ophiocordyceps rubroflava* is described as a new species distinguished by stromata arising from the host’s back, immersed perithecia, and cylindrical part-spores. Both taxa form well-supported independent lineages within the *O. ravenelii* clade, closely related to *O. formosana*, *O. melolonthae* and *O. neovolkiana*. These findings expand the known diversity and host range of the *O. ravenelii* complex in East Asia and underscore the need for continued surveys of Coleopteran-associated *Ophiocordyceps* in under-explored regions.

## Introduction

The clavicipitoid fungi (Hypocreales), comprising Clavicipitaceae, Cordycipitaceae, Ophiocordycipitaceae, and Polycephalomycetaceae, represent an ecologically significant group closely associated with arthropods (Araújo et al. 2018; Xiao et al. 2023; Dong et al. 2025; Mongkolsamrit et al. 2025). Among these, the Ophiocordycipitaceae stands out for its remarkable diversity, harboring fungi of substantial ecological, economic, and medicinal importance (Dai et al. 2026). The type genus, *Ophiocordyceps* Petch, was originally erected to accommodate *Cordyceps*-like fungi with clavate asci and non-disarticulating ascospores (Petch 1931). Since the establishment of the genus by Petch in 1931, many mycologists have contributed to its taxonomy based on morphological criteria (Kobayasi 1941; Mains 1958; Kobayasi 1981; Evans and Samson 1984; Zang and Kinjo 1998; Li et al. 2005; Liang et al. 2007). This morphology-based concept was subsequently refined with the advent of molecular phylogenetics. Sung et al. (2007a) resurrected and emended *Ophiocordyceps* using multi-locus data (nrSSU, nrLSU, *tef1-α*, *rpb1*, and *rpb2*), solidifying its position within a redefined Ophiocordycipitaceae and broadening its circumscription. Subsequent molecular phylogenetic studies have further advanced its taxonomy (Quandt et al. 2014; Spatafora et al. 2015; Araújo et al. 2018; Khonsanit et al. 2018; Xu et al. 2022). *Ophiocordyceps* is the largest and most speciose genus within Ophiocordycipitaceae, currently comprising more than 373 accepted names that represent approximately 70% of the family’s described diversity (https://www.indexfungorum.org, accessed on March 15, 2026).

Morphologically, *Ophiocordyceps* exhibits remarkable diversity. The sexual morph is characterized by stromata ranging from dark and wiry to brightly colored and fleshy, perithecia that may be superficial or immersed, and ascospores that either remain intact or disarticulate into part-spores (Sung et al. 2007a; Luangsa-Ard et al. 2018). The genus is holomorphic, with asexual morphs most commonly of the *Hirsutella*-type, although *Hymenostilbe*, *Paraisaria*, and *Syngliocladium* morphs are also documented (Quandt et al. 2014; Ban et al. 2015; Araújo et al. 2018; Khonsanit et al. 2018; Xie et al. 2025). *Hymenostilbe* morphs are particularly frequent among ant-associated lineages (Evans and Samson 1984; Araújo et al. 2018). This morphological plasticity, coupled with a broad host range spanning at least nine insect orders (Blattodea, Coleoptera, Diptera, Hemiptera, Hymenoptera (especially ants), Lepidoptera, Megaloptera, Neuroptera, Odonata), has made *Ophiocordyceps* a focal group for ecological and evolutionary research (Tasanathai et al. 2020; Thanakitpipattana et al. 2020; Wei et al. 2020; Wang et al. 2021; Zha et al. 2021; Sun et al. 2022; Tang et al. 2022; Tasanathai et al. 2022; Tian et al. 2025). Furthermore, several species, most notably *O. sinensis* and *O. sobolifera*, are renowned for their use in traditional medicine, driving considerable interest in their biology and chemistry (Luangsa-Ard et al. 2018; Zhou et al. 2022).

Phylogenetic analyses have resolved *Ophiocordyceps* into major clades, including *O. ravenelii*, *O. sphecocephala*, *O. sobolifera*, and a large *Hirsutella* clade (Sanjuan et al. 2015; Wang et al. 2018; Zou et al. 2022). The *O. ravenelii* clade predominantly parasitizes Coleoptera larvae, especially Scarabaeidae, producing yellow to brown wiry stromata with immersed perithecia, and is characterized by filamentous, multiseptate ascospores that disarticulate into cylindrical part-spores (Li et al. 2005; Sanjuan et al. 2015; Yang et al. 2015; Zhang et al. 2026). Its asexual morph features phialides borne singly, oppositely, or in whorls, with swollen bases tapering into slender necks, and typically lacks mucus sheaths (Li et al. 2005; Wang et al. 2015). Key members include *O. barnesii*, *O. gracillima*, *O. highlandensis*, *O. konnoana*, *O. melolonthae*, and *O. ravenelii* (Kobayasi 1941; Sanjuan et al. 2015; Yang et al. 2015; Mongkolsamrit et al. 2024; Zhang et al. 2026). Despite its phylogenetic distinctiveness, this lineage remains understudied compared to the *O. unilateralis* complex, and diversity associated with non-scarabaeid beetles (Elateroidea) is largely unexplored, offering potential insights into host range evolution and biogeography.

Based on specimens collected from coleopteran larvae in Yunnan Province, China, we provide an emended description of *O. jinguangensis* and describe *O. rubroflava* as a new species. Their morphology and multi-locus phylogeny support their recognition as distinct taxa within *Ophiocordyceps*. This study contributes to the known diversity of entomopathogenic fungi associated with beetles in Southwest China.

## Materials and methods

### Specimen collection

Five fungal specimens were collected from Yunnan, China, photographed in the field, and documented with site, GPS coordinates, and altitude. Surface debris was removed in the field using a soft brush. Samples were then placed in sterile containers and transported to the laboratory at 4 °C, where they were washed with sterile water, assigned accession numbers CXAC 0021–0025, and air-dried. Voucher specimens are deposited at the College of Agronomy Herbarium (**CXAC**), Chuxiong Normal University, China, and are available for taxonomic identification, molecular analysis, and future studies.

### Morphological characterization

Specimen data included host associations and geographical origin. For morphological study, sexual morph structures (perithecia, asci, ascospores, part-spores) were mounted in lactophenol cotton blue solution. These structures were observed and measured using an Olympus BX53 compound microscope (Olympus Corporation, Tokyo, Japan). Twenty to 50 measurements were taken for each type of structure; the range and standard deviation were subsequently calculated.

### Extraction of DNA, polymerase chain reaction (PCR), and molecular sequencing

Genomic DNA was extracted from fresh fungal tissue using a commercial plant DNA isolation kit (FOREGENE, China), following the manufacturer’s protocol. Five genetic regions (nrSSU, nrLSU, *tef1-α*, *rpb1*, and *rpb2*) were amplified following Dong et al. (2026). Each 25 μL PCR reaction contained 1× PCR buffer (final Mg^2+^ concentration 2 mM), 0.2 mM dNTPs, 0.4 μM of each primer, 0.625 U *Taq* DNA polymerase, and 1 μL DNA template. PCR amplifications were performed using the primer pairs and touchdown thermal cycling program described in Dong et al. (2026), with an initial denaturation at 95 °C for 4 min, followed by touchdown and standard cycling, and a final extension at 72 °C for 8 min. PCR products were verified by gel electrophoresis, purified, and sent to the Beijing Genomics Institute (Shenzhen, China) for Sanger dideoxy sequencing with the same primer pairs.

### Phylogenetic analyses

To reconstruct phylogenetic relationships, a five-locus dataset was assembled, comprising nrSSU, nrLSU, *tef1-α*, *rpb1*, and *rpb2*. Newly generated sequences were validated using BLAST searches against GenBank and combined with reference sequences from related taxa (Table 1). Multiple sequence alignments were generated and manually edited using MUSCLE implemented in MEGA X (Kumar et al. 2018). Phylogenetic analyses were conducted using the maximum likelihood (ML) and Bayesian inference (BI). ML analysis used IQ-TREE v3.1.1 with partitioning and model selection by ModelFinder (Kalyaanamoorthy et al. 2017) under BIC. The best-fit models were: nrSSU = TPM3u+I+R4; nrLSU = TIM+F+R5; *tef1-α* = GTR+F+I+R6; *rpb1* = TIM+F+I+R4; *rpb2* = TIM3+F+R4. Branch support was evaluated with 1,000 ultrafast bootstrap replicates (Nguyen et al. 2015). Bayesian inference (BI) was conducted with MrBayes v3.2.7 using the best-fit model (GTR+G), as determined by MrModeltest v2.2 (Nylander 2004). Markov chain Monte Carlo (MCMC) sampling was performed for 5,000,000 generations, with trees sampled every 1,000 generations. Convergence was checked in Tracer v1.7.2, ensuring all effective sample sizes (ESS) >200; the first 25% of trees were discarded as burn-in. Runs were terminated once the average standard deviation of split frequencies fell below 0.01. The resulting trees were visualized and annotated following Xie et al. (2023) and the layout was finalized in Adobe Illustrator CS6.

**Table 1.** Specimen information and GenBank accession numbers for sequences used in this study.

Current name	Voucher	GenBank accession number					References
		nrSSU	nrLSU	*tef1-α*	*rpb1*	*rpb2*	
*Hirsutella cf. haptospora*	ARSEF 2228	KM652075	KM652118	KM652001	KM652041	–	Simmons et al. 2015
*H. citriformis*	ARSEF 1446	KM652065	KM652106	KM651990	KM652031	–	Simmons et al. 2015
*H. cryptosclerotium*	ARSEF 4517	KM652066	KM652109	KM651992	KM652032	–	Simmons et al. 2015
*H. fusiformis*	ARSEF 5474	KM652067	KM652110	KM651993	KM652033	–	Simmons et al. 2015
*H. gigantea*	ARSEF 30	–	JX566977	JX566980	KM652034	–	Simmons et al. 2015
*H. guyana*	ARSEF 878	KM652068	KM652111	KM651994	KM652035	–	Simmons et al. 2015
*H. haptospora*	ARSEF 2226	–	–	KM651995	KM652036	–	Simmons et al. 2015
*H. illustris*	ARSEF 5539	KM652069	KM652112	KM651996	KM652037	–	Simmons et al. 2015
*H. kirchneri*	ARSEF 5551	KM652070	KM652113	KM651997	–	–	Simmons et al. 2015
*H. lecaniicola*	ARSEF 8888	KM652071	KM652114	KM651998	KM652038	–	Simmons et al. 2015
*H. liboensis*	ARSEF 9603	KM652072	KM652115	–	–	–	Simmons et al. 2015
*H. necatrix*	ARSEF 5549	KM652073	KM652116	KM651999	KM652039	–	Simmons et al. 2015
*H. nodulosa*	ARSEF 5473	KM652074	KM652117	KM652000	KM652040	–	Simmons et al. 2015
*H. radiata*	ARSEF 1369	KM652076	KM652119	KM652002	KM652042	–	Simmons et al. 2015
*H. rhossiliensis*	ARSEF 3747	KM652080	KM652123	KM652006	KM652045	–	Simmons et al. 2015
*H. satumaensis*	ARSEF 996	KM652082	KM652125	KM652008	KM652047	–	Simmons et al. 2015
*H. strigosa*	ARSEF 2197	KM652085	KM652129	KM652012	KM652050	–	Simmons et al. 2015
*H. subulata*	ARSEF 2227	KM652086	KM652130	KM652013	KM652051	–	Simmons et al. 2015
*H. thompsonii*	ARSEF 414	–	KM652143	KM652021	KM652059	–	Simmons et al. 2015
*H. thompsonii* var. *vina*	ARSEF 254	KM652101	KM652149	KM652028	KM652062	–	Simmons et al. 2015
*H. versicolor*	ARSEF 1037	KM652102	KM652150	KM652029	KM652063	–	Simmons et al. 2015
*Ophiocordyceps acicularis*	OSC 110988	EF468951	EF468804	EF468745	EF468853	–	Sung et al. 2007a
*O. agriotidis*	ARSEF 5692	DQ522540	DQ518754	DQ522322	DQ522368	DQ522418	Spatafora et al. 2007
*O. appendiculata*	NBRC 106960	JN941728	JN941413	AB968577	JN992462	AB968539	Schoch et al. 2012
*O. arborescens*	NBRC 105891	AB968386	AB968414	AB968572	–	AB968534	Ban et al. 2015
*O. asiana*	MY11878	–	MW280213	MW292448	MW296049	–	Khao-ngam et al. 2021
*O. asiatica*	BCC 30516	–	MH753675	MK284263	MK214105	MK214091	Tasanathai et al. 2019
*O. barnesii*	BCC 28560	EU408776	–	–	EU408773	EU418599	Luangsa-Ard et al. 2010
*O. bidoupensis*	YFCC 8793	OM304638	–	OK556894	OK556898	OK556900	Zou et al. 2022
*O. brunneinigra*	BCC 69032	–	MF614654	MF614638	MF614668	MF614681	Luangsa-Ard et al. 2018
*O. brunneiperitheciata*	BCC 66167	–	MF614659	MF614644	–	MF614684	Luangsa-Ard et al. 2018
*O. brunneipunctata*	OSC 128576	DQ522542	DQ518756	DQ522324	DQ522369	DQ522420	Spatafora et al. 2007
*O. brunneirubra*	BCC 14384	–	MH753690	GU797121	MK751465	MK751468	Tasanathai et al. 2019
*O. campes*	BCC 36938	–	MT118175	MT118167	MT118183	MT118188	Tasanathai et al. 2020
*O. clavata*	NBRC 106961	JN941727	JN941414	AB968586	JN992461	AB968547	Sung et al. 2007a
*O. clavata*	CEM 1762	KJ878916	KJ878882	KJ878963	KJ878996	–	Quandt et al. 2014
*O. communis*	BCC 1842	–	MH753680	MK284266	MK214110	MK214096	Tasanathai et al. 2019
*O. cossidarum*	MFLU 17-0752	MF398186	MF398187	MF928403	MF928404	–	Xiao et al. 2019
*O. crinalis*	GDGM 17327	KF226253	KF226254	KF226256	KF226255	–	Wang et al. 2014
*O. dipterigena*	OSC 151911	KJ878919	KJ878886	KJ878966	KJ879000	–	Quandt et al. 2014
*O. elongata*	OSC 110989	–	EF468808	EF468748	EF468856	–	Sung et al. 2007a
*O. flavida*	BCC 84256	–	MT512655	MT533482	MT533476	–	Mongkolsamrit et al. 2021
*O. formosana*	TNM F13893	KJ878908	–	KJ878956	KJ878988	KJ878943	Quandt et al. 2014
*O. furcatosubulata*	YFCC 902	MT774214	MT774221	MT774242	MT774228	MT774235	Wang et al. 2021
*O. fusiformis*	BCC 93025	–	MZ675422	MZ707849	MZ707855	MZ707805	Tasanathai et al. 2022
*O. fusiformis*	BCC 93026	–	MZ675423	MZ707850	MZ707856	MZ707806	Tasanathai et al. 2022
*O. geometridicola*	BCC 35947	–	MF614647	MF614631	MF614664	MF614678	Luangsa-Ard et al. 2018
*O. globiceps*	MFLU 18-0661	MH725812	MH725830	MH727388	–	–	Xiao et al. 2019
*O. globiperitheciata*	HKAS 126130	OR082950	OR015968	OR030532	OR119834	–	Fan et al. 2024
*O. globiperitheciata*	HKAS 126131	OR082951	OR015969	OR030533	OR119835	–	Fan et al. 2024
*O. globosa*	BCC 93023	–	MZ675419	MZ707846	MZ707861	–	Tasanathai et al. 2022
*O. halabalaensis*	MY5151	KM655826	–	GU797110	–	–	Luangsa-Ard et al. 2011
*O. highlandensis*	HKAS83207	KM581284	–	–	KM581274	KM581278	Yang et al. 2015
*O. hydrangea*	YFCC 8832	OM304636	OM304640	OM831277	OM831280	OM831283	Zou et al. 2022
*O. irangiensis*	BCC 82795	–	–	MH028186	MH028164	MH028174	Tasanathai et al. 2019
*O. isopterae*	MY12376	–	MZ675420	MZ707847	MZ707859	MZ707803	Tasanathai et al. 2022
*O. isopterae*	BCC 93042	–	MZ675421	MZ707848	–	MZ707804	Tasanathai et al. 2022
*O. jinguangensis*	**CXAC 0024**	**PX973455**	**PX973460**	**PX984913**	**PX990088**	**PX984942**	**This study**
*O. jinguangensis*	**CXAC 0025**	**PX973456**	**PX973461**	**PX984914**	**PX990089**	**PX984943**	**This study**
*O. jinguangensis*	HKAS 149981	PX692985	–	PX694736	PX694717	PX694727	Zhang et al. 2026
*O. jinguangensis*	HKAS 149982	PX692986	–	PX694737	PX694718	PX694728	Zhang et al. 2026
*O. karstii*	MFLU 15-3884	KU854952	–	KU854945	KU854943	–	Li et al. 2016
*O. khokpasiensis*	BCC 48071	–	MH753682	MK284269	MK214112	–	Tasanathai et al. 2019
*O. kimflemingiae*	SC09B	KX713631	KX713620	KX713698	KX713724	–	Araújo et al. 2018
*O. konnoana*	EFCC 7315	EF468959	–	EF468753	EF468861	EF468916	Sung et al. 2007a
*O. krachonicola*	BCC79666	–	MK632080	MK632054	MK632161	MK632132	Thanakitpipattana et al. 2020
*O. kuchinaraiensis*	BCC 95830	–	OQ627397	OQ625474	–	OQ625475	Tasanathai et al. 2019
*O. linyphiidarum*	HKAS 132197	PV139229	PV139245	PV156008	PV155979	PV155994	Yang et al. 2025
*O. linyphiidarum*	HKAS 132196	PV139230	PV139246	PV156009	PV155980	PV155995	Yang et al. 2025
*O. longissima*	EFCC 6814	–	EF468817	EF468757	EF468865	–	Sung et al. 2007a
*O. longissima*	NBRC 106965	AB968392	AB968420	AB968584	–	AB968546	Ban et al. 2015
*O. longistipes*	KUNCC 5224	OR082949	OR015967	OR030530	OR062224	OR113082	Fan et al. 2024
*O. longistromata*	BCC 44497	–	MT118178	MT118170	–	MT118191	Tasanathai et al. 2020
*O. macroacicularis*	NBRC 100685	AB968388	AB968416	AB968574	–	AB968536	Ban et al. 2015
*O. megacuculla*	BCC 82984	–	MH028162	MH028192	–	MH028181	Tasanathai et al. 2019
*O. melolonthae*	OSC 110993	DQ522548	DQ518762	DQ522331	DQ522376	–	Mongkolsamrit et al. 2019
*O. mosingtoensis*	BCC 30904	–	MH753686	MK284273	MK214115	MK214100	Tasanathai et al. 2019
*O. mosingtoensis*	BCC 36921	–	MH753685	MK284272	MK214116	MK214099	Tasanathai et al. 2019
*O. multiperitheciata*	BCC 22861	–	MF614656	MF614640	MF614670	MF614683	Luangsa-Ard et al. 2018
*O. myrmecophila*	CEM 1710	–	KJ878894	KJ878974	KJ879008	–	Quandt et al. 2014
*O. neovolkiana*	OSC 151903	KJ878930	KJ878896	KJ878976	KJ879010	–	Quandt et al. 2014
*O. nigrella*	EFCC 9247	EF468963	EF468818	EF468758	EF468866	EF468920	Sung et al. 2007a
*O. nutans*	OSC 110994	DQ522549	DQ518763	DQ522333	DQ522378	–	Spatafora et al. 2007
*O. ovatospora*	YHH 2206001	OP295110	OP295113	OP313801	OP313803	OP313805	Tang et al. 2022
*O. ovatospora*	YFCC 22069184	OP295111	OP295114	OP313802	OP313804	–	Tang et al. 2022
*O. pauciovoperitheciata*	TBRC 8096	–	MF614649	MF614636	MF614665	MF614672	Luangsa-Ard et al. 2018
*O. phuwiangensis*	BCC 85351	–	–	MT118174	MT118187	MT118195	Tasanathai et al. 2020
*O. pruinosa*	NHJ 12994	EU369106	EU369041	EU369024	EU369063	EU369084	Johnson et al. 2009
*O. pseudoacicularis*	BCC 53843	–	MF614646	MF614630	MF614661	MF614677	Luangsa-Ard et al. 2018
*O. pseudocommunis*	BCC 16757	–	MH753687	MK284274	MK214117	MK214101	Tasanathai et al. 2019
*O. pseudolloydii*	MFLUCC 15-0689	–	–	MF372758	MF372761	–	Xiao et al. 2017
*O. pseudorhizoidea*	BCC 86431	–	MH753674	MK284262	MK751469	MK214090	Tasanathai et al. 2019
*O. pseudovariabilis*	BCC 88308	–	–	OR855799	OR855823	–	Mongkolsamrit et al. 2024
*O. puluongensis*	YFCC 6442	MT141118	MT270528	MT270520	MT270523	MT270526	Xu et al. 2022
*O. pulvinata*	TNS-F 30044	GU904208	–	GU904209	GU904210	–	Kepler et al. 2011
*O. purpureostromata*	TNS F18430	KJ878931	KJ878897	KJ878977	KJ879011	–	Quandt et al. 2014
*O. radiciformis*	BCC 93036	–	MZ675425	MZ707852	MZ707857	MZ707808	Tasanathai et al. 2022
*O. radiciformis*	BCC 93035	–	MZ675426	MZ707853	MZ707858	MZ707809	Tasanathai et al. 2022
*O. ramosissimum*	GZUHHN8	KJ028012	–	KJ028014	KJ028017	–	Wen et al. 2014
*O. ravenelii*	OSC 110995	DQ522550	DQ518764	DQ522334	DQ522379	DQ522430	Spatafora et al. 2007
*O. ravenelii*	OSC 151914	KJ878932	–	KJ878978	KJ879012	KJ878950	Quandt et al. 2014
*O. rhizoidea*	NHJ 12522	EF468970	EF468825	EF468764	EF468873	EF468923	Tasanathai et al. 2019
*O. rhizoidea*	NHJ 12529	EF468969	EF468824	EF468765	EF468872	EF468922	Tasanathai et al. 2019
*O. robertsii*	KEW 27083	–	EF468826	EF468766	–	–	Sung et al. 2007a
*O. rubiginosiperitheciata*	NBRC 106966	JN941704	JN941437	AB968582	JN992438	AB968544	Kepler et al. 2012
*O. rubroflava*	**CXAC 0021**	**PX973452**	**PX973457**	**PX984910**	**PX990085**	**PX984939**	**This study**
*O. rubroflava*	**CXAC 0022**	**PX973453**	**PX973458**	**PX984911**	**PX990086**	**PX984940**	**This study**
*O. rubroflava*	**CXAC 0023**	**PX973454**	**PX973459**	**PX984912**	**PX990087**	**PX984941**	**This study**
*O. salganeicola*	Mori01	MT741705	MT741719	MT759575	MT759578	MT759580	Araujo et al. 2021
*O. salganeicola*	Mori02	MT741704	MT741718	MT759572	MT759579	MT759581	Araujo et al. 2021
*O. satoi*	J7	KX713653	KX713599	KX713683	KX713711	–	Araújo et al. 2018
*O. sinensis*	ARSEF 6282	KM652083	KM652126	KM652009	KM652048	–	Simmons et al. 2015
*O. sinensis*	EFCC 7287	EF468971	EF468827	EF468767	EF468874	EF468924	Kepler et al. 2012
*O. sobolifera*	NBRC 106967	AB968395	AB968422	AB968590	–	–	Ban et al. 2015
*O. spataforae*	NHJ 12525	EF469125	EF469078	EF469063	EF469092	EF469111	Sung et al. 2007a
*O. spataforae*	OSC 128575	EF469126	EF469079	EF469064	EF469093	EF469110	Sung et al. 2007a
*O. sphecocephala*	NBRC 101752	JN941696	JN941445	AB968591	JN992430	AB968552	Schoch et al. 2012
*O. stylophora*	OSC 111000	DQ522552	DQ518766	DQ522337	DQ522382	DQ522433	Spatafora et al. 2007
*O. stylophora*	OSC 110999	EF468982	EF468837	–	EF468882	EF468931	Sung et al. 2007a
*O. superficialis*	MICH 36253	EF468983	–	–	EF468883	–	Sung et al. 2007a
*O. termiticola*	BCC 1920	–	MH753678	MK284265	MK214108	MK214094	Tasanathai et al. 2019
*O. thanathonensis*	MFLU 16-2909	–	MF850377	MF872613	MF872615	–	Xiao et al. 2017
*O. tricentri*	NBRC 106968	AB968393	AB968423	AB968593	–	AB968554	Ban et al. 2015
*O. trichospora*	CBS 109876	AF543766	AF543790	AF543779	AY489669	DQ522457	Sung et al. 2007a
*O. unilateralis*	OSC 128574	DQ522554	DQ518768	DQ522339	DQ522385	DQ522436	Spatafora et al. 2007
*O. unituberculata*	YFCC HU1301	KY923214	–	KY923216	KY923218	KY923220	Wang et al. 2018
*O. variabilis*	ARSEF 5365	DQ522555	DQ518769	DQ522340	DQ522386	–	Sung et al. 2007a
*O. variabilis*	OSC 111003	EF468985	EF468839	EF468779	EF468885	EF468933	Sung et al. 2007b
*O. xuefengensis*	GZUH2012HN14	KC631789	–	KC631793	KC631798	–	Wen et al. 2013
*Tolypocladium inflatum*	OSC 71235	EF469124	EF469077	EF469061	EF469090	EF469108	Sung et al. 2007b
*T. ophioglossoides*	CBS 100239	KJ878910	KJ878874	KJ878958	KJ878990	KJ878944	Quandt et al. 2014

Boldface: data generated in this study, ^T^ ex-type material, – means no data.

## Results

### Phylogenetic analyses

Phylogenetic relationships within *Ophiocordyceps* were reconstructed based on an alignment of 131 taxa (Table 1). *Tolypocladium inflatum* OSC 71235 and *T. ophioglossoides* CBS 100239 were used as the outgroups. The final concatenated matrix consisted of 5,156 bp (including gaps), partitioned as follows: nrSSU 1,488 bp, nrLSU 926 bp, *tef1-α* 962 bp, *rpb1* 718 bp, and *rpb2* 1,062 bp. Both BI and ML analyses generated highly congruent topologies.

**Figure 1.**
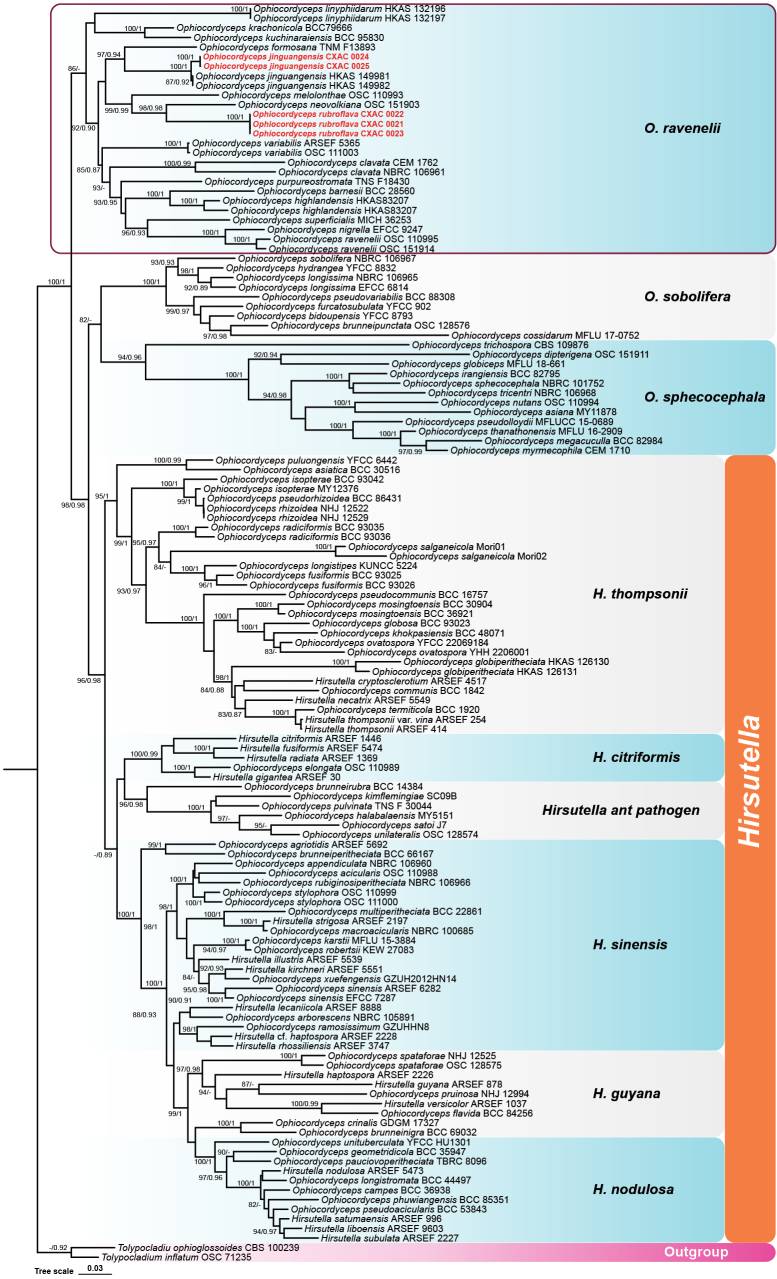
Molecular phylogenetic analyses using the ML and BI based on combined nrSSU, nrLSU, *tef1-α*, *rpb1*, and *rpb2* sequence data. *Tolypocladium inflatum* OSC 71235 and *T. ophioglossoides* CBS 100239 were used as outgroups. Statistical support values (BS ≥ 80% and PP ≥ 0.80) are shown at the nodes for ML bootstrap support (BS) and BI posterior probabilities (PP), nodes without values received support below these thresholds. Isolates in red type are those analyzed in this study. The scale bar represents the expected number of changes per site.

The genus *Ophiocordyceps* was resolved into four well-supported major clades (Fig. 1), designated here as: the *O. ravenelii* clade, the *O. sobolifera* clade, the *O. sphecocephala* clade and *Hirsutella* clade. The *Hirsutella* clade further comprised six subclades: the *H. thompsonii* subclade, *H. citriformis* subclade, *Hirsutella* ant pathogen subclade, *H. sinensis* subclade, *H. guyana* subclade, and *H. nodulosa* subclade. The present study focuses on the *O. ravenelii* clade, which corresponds to the *O. ravenelii* species complex *sensu lato*.

The *O. ravenelii* clade received strong support and comprised *Ophiocordyceps barnesii*, *O. clavata*, *O. formosana*, *O. highlandensis*, *O. jinguangensis*, *O. krachonicola*, *O. kuchinaraiensis*, *O. linyphiidarum*, *O. melolonthae*, *O. neovolkiana*, *O. nigrella*, *O. purpureostromata*, *O. ravenelii*, *O. superficialis*, *O. variabilis*, and the new species *O. rubroflava* (Fig. 1). Our specimens CXAC 0024 and CXAC 0025 clustered with the recently described *O. jinguangensis* (HKAS 149981 and HKAS 149982) with maximal support, and the pairwise genetic distances among them across the four loci (nrSSU, *tef1-α*, *rpb1*, and *rpb2*) were negligible (≤0.2%). These specimens are therefore identified as *O. jinguangensis*. Our collection represents the first report of this species from Xinping County, Yunnan Province, and expands its known host range to include Elateroidea larvae. The new species *O. rubroflava*, represented by specimens CXAC 0021, CXAC 0022, and CXAC 0023, formed a distinct, strongly supported lineage sister to *O. formosana* and *O. neovolkiana*, clearly separated from all other members of the clade.

### Morphological features

**Figure 2.**
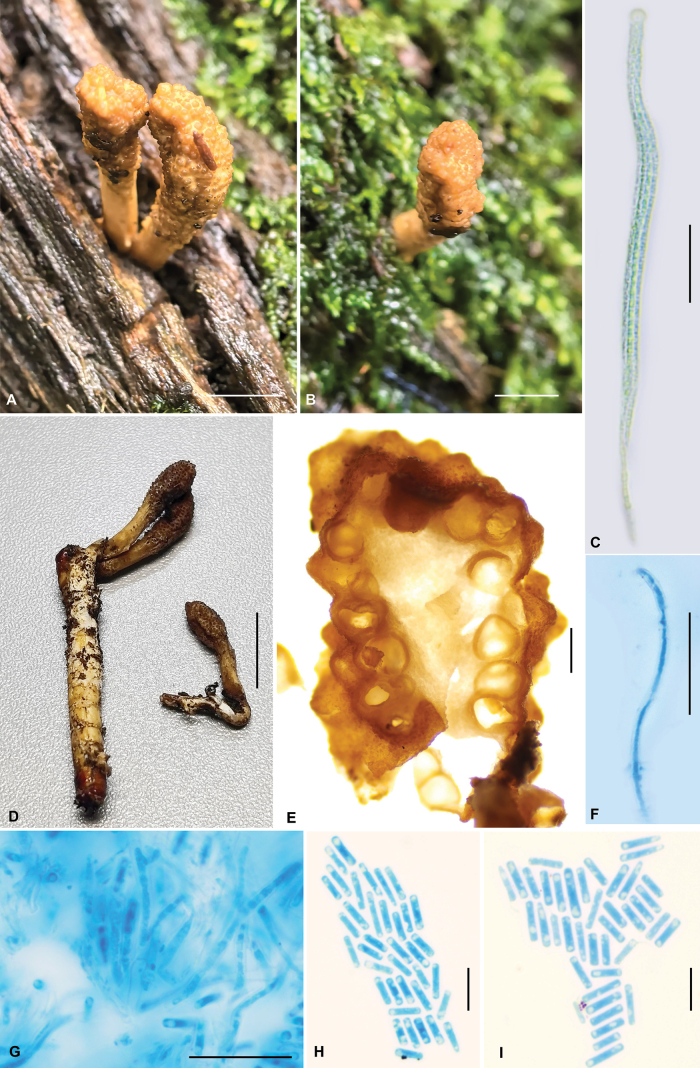
Morphological features of *Ophiocordyceps jinguangensis* (CXAC 0024). **A, B, D**. Fungus on Elateroidea larvae; **C**. Asci; **E**. Perithecia; **F, G**. Ascospore segments; **H, I**. Part-spores. Scale bars: 5 mm (**A, B**); 50 µm (**C**); 10 mm (**D**); 500 µm (**E**); 20 µm (**F, G**); 10 µm (**H, I**).

**Figure 3.**
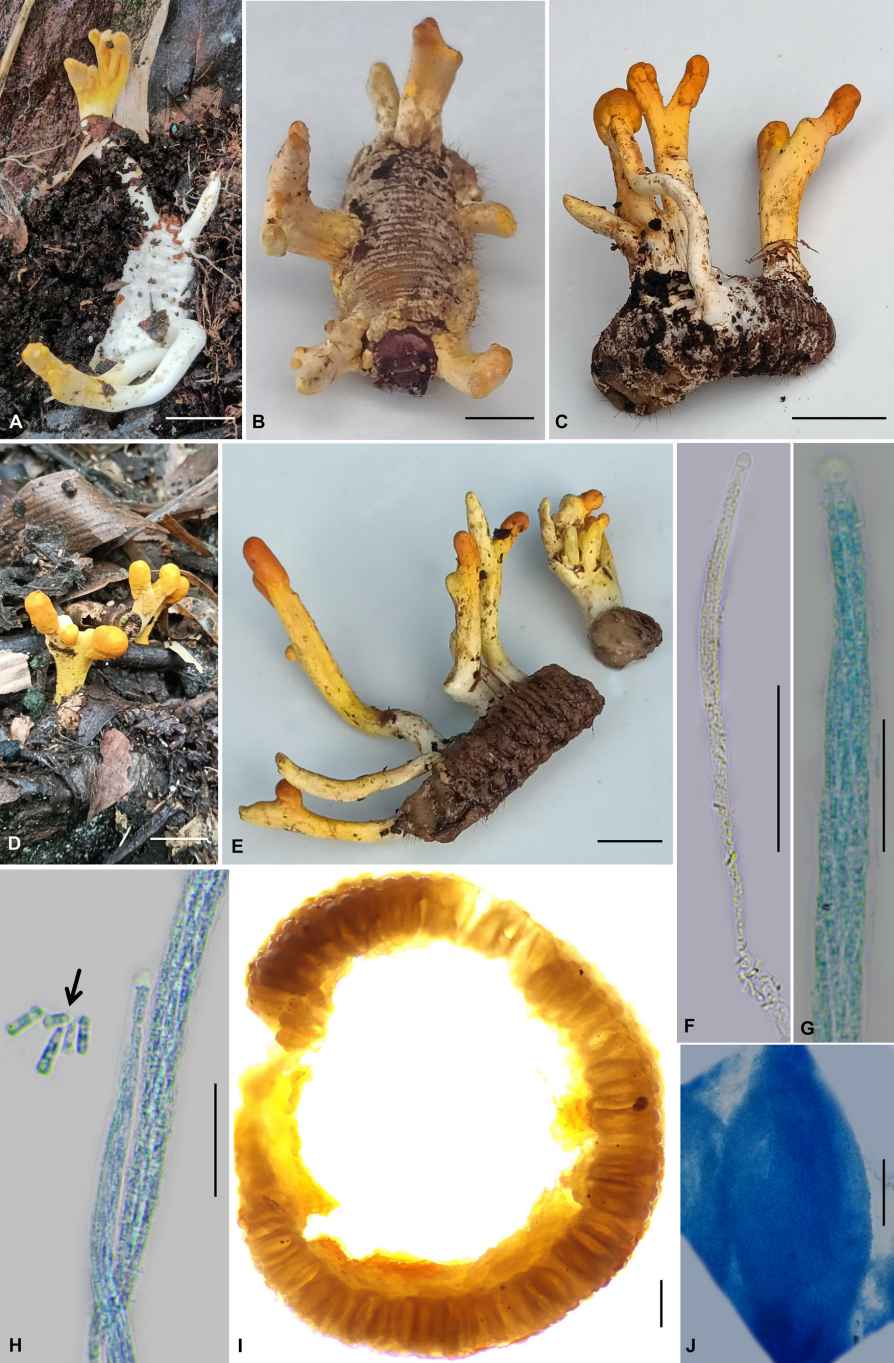
Morphological features of *Ophiocordyceps rubroflava* (holotype CXAC 0021). **A–E**. Fungus on Coleoptera larva; **F, G**. Ascus; **H**. Asci and part-spores (arrow); **I, J**. Perithecia. Scale bars: 10 mm (**A–E**); 50 µm (**F**); 20 µm (**G, H**); 500 µm (**I**); 200 µm (**J**);

The morphological characteristics and photomicrographs of the two species studied, *Ophiocordyceps jinguangensis* and *Ophiocordyceps rubroflava*, are presented in Figs 2, 3, with comprehensive descriptions provided in the Taxonomy section.

### Taxonomy

Two species are described in this study.

#### Ophiocordyceps jinguangensis

Taxon classificationFungiHypocrealesOphiocordycipitaceae

X. Zhang, C.J.Y. Li, K.D. Hyde & T.C. Wen, IMA Fungus 17: e171084, 17 (2026)

E8F2FB95-0BB9-539F-B085-51C86408E42B

Fig. 2

##### Sexual morph.

***Stromata*** arising from the head of Elateroidea buried in decaying wood, solitary or in pairs, unbranched, cylindrical, fleshy, yellow to yellowish brown, 12–25 mm long. ***Fertile parts*** clavate, pale yellow to yellowish brown, 7–15 mm long, 2.5–4.5 mm wide, generating toward the upper part of stromata, covered by a verrucose surface. ***Stipe*** pale yellow to brown, 0.5–2.5 mm wide. ***Perithecia*** immersed, ovoid to oblong-ovate, densely distributed in the upper of stromata, arranged in a disordered manner, 283–550 × 175–375 µm. ***Asci*** 8-spored, filiform, hyaline, 235–355 × 6.5–7.5 µm, with hemispheric apical cap, 4.0–5.5 µm high. ***Ascospores*** filiform, multi-septate, 210–305 × 1.5–2.0 µm. ***Part-spores*** cylindrical, 4.5–8.0 × 1.0–2.5 µm. **Asexual morph**: Undetermined.

##### Material examined.

China, • Yunnan Province, Yuxi City, Xinping Yi and Dai Autonomous County, Elateroidea larvae (Coleoptera), 9 August 2025, Quanying Dong, CXAC 0024, CXAC 0025.

##### Host.

Elateroidea larvae (Coleoptera).

##### Habitat.

On Elateroidea larvae buried in decaying wood.

##### Known distribution.

Yunnan Province, China.

##### Commentary.

Specimens CXAC 0024 and CXAC 0025 were identified as *Ophiocordyceps jinguangensis* based on multi-locus phylogenetic analysis and negligible genetic distances (≤0.2% across four loci). Our material expands the known host range to include Elateroidea larvae (vs. Tenebrionoidea in the type material), and extends the morphological range to longer asci (235–355 μm vs 153.6–273.2 μm) and wider perithecia (175–375 μm vs 134–223 μm).

In our multi-locus phylogeny (Fig. 1), *O. jinguangensis* nests within the *O. ravenelii* clade and is closely related to *O. formosana*, *O. melolonthae*, *O. neovolkiana* and *O. rubroflava*. *Ophiocordyceps jinguangensis* resembles *O. formosana* in producing simple or occasionally branched stromata from Coleopteran larvae in decaying wood, but *O. formosana* differs by its branched stromata, Tenebrionidae host, semi-immersed ovate perithecia (453–546 × 265–298 μm) and shorter part-spores (2–6 × 1–3 μm) (Kobayasi and Shimizu 1981; Shimizu 1994; Li et al. 2005; Li et al. 2016).

*Ophiocordyceps melolonthae* (Mains 1958) differs from *O. jinguangensis* in its substantially longer stromata (3–13 cm vs 1.2–2.5 cm), sulphur-yellow to mikado-orange coloration, association with Scarabaeidae larvae vs Elateroidea, narrower part-spores (1–1.5 µm vs 1.0–2.5 µm), and smaller perithecia (170–264 µm vs 175–375 µm wide).

*Ophiocordyceps neovolkiana*, another related species, differs from *O. jinguangensis* both in host association and morphology. Originally described from Japan by Kobayasi (1941) and subsequently reported from India and Vietnam (Giang et al. 2015; Laya et al. 2019), *O. neovolkiana* is characterized by an ovoid or depressed-sphaeroid or tuberiform fertile part. Additionally, it possesses smaller perithecia (340–460 × 140–165 µm vs 283–550 × 175–375 µm) and shorter asci (230–300 × 9–10 µm vs 235–355 × 6.5–7.5 µm) (Kobayasi 1941).

*Ophiocordyceps rubroflava*, described herein as a new species, differs from *O. jinguangensis* in its longer (3–4.5 cm), bright orange to yellow-orange stromata, clavate to spherical fertile part, and smaller perithecia (275–380 × 105–180 µm vs 283–550 × 175–375 µm), shorter asci (160–195 × 4.5–6.5 µm vs 235–355 × 6.5–7.5 µm), and smaller part-spores (3.0–6.0 × 1.0–2.0 µm vs 4.5–8.0 × 1.0–2.5 µm).

#### Ophiocordyceps rubroflava

Taxon classificationFungiHypocrealesOphiocordycipitaceae

Q.Y. Dong & C. D. Xu sp. nov.

7FE90A59-4B96-53BF-8F82-29CEB28C5182

862339

Fig. 3

##### Etymology.

*rubroflava*, from *rubro*- = red-, *ﬂava* = yellow, referring to its red fertile part and yellow stipe of stromata.

##### Holotype.

China, • Yunnan Province, Pu’er City, Lancang Lahu Autonomous County, On the larva of Coleoptera, 28 August 2025, Quanying Dong (holotype CXAC 0021).

##### Sexual morph.

***Stromata*** arising from the back of Coleoptera buried in soil, sometimes the host covered by a white mycelial layer, solitary or in groups of 2–8, cylindrical, soft, fleshy, 3–4.5 cm long, 3–8 mm wide, bright orange to yellow orange, either branched or unbranched. ***Stipe*** cylindrical, bright orange or white, with the base near the host body typically white, 2.5–4 cm long. ***Fertile parts*** clavate or spherical, red to orange red, 3–10 mm long, occupying the upper part of stromata. ***Perithecia*** immersed, bright yellow when young, becoming yellowish brown at maturity, ovoid to oblong-ovate, densely distributed in a granular pattern on the upper part of the stromata, 275–380 × 105–180 µm. ***Asci*** 8-spored, filiform, hyaline, 160–195 × 4.5–6.5 µm, with a hemispherical cap, 4.5–5.5 µm high. ***Part-spores*** cylindrical, 3.0–6.0 × 1.0–2.0 µm. **Asexual morph**: Undetermined.

##### Host.

Coleoptera larva

##### Habitat.

On Coleoptera larvae buried in soil.

##### Known distribution.

Yunnan Province, China.

##### Additional specimens examined.

China, • Yunnan Province, Pu’er City, Lancang Lahu Autonomous County, On the larva of Coleoptera, 28 August 2025, Quanying Dong (paratype CXAC 0022). China, • Yunnan Province, Pu’er City, Lancang Lahu Autonomous County, On the larva of Coleoptera, 29 August 2025, Quanying Dong (paratype CXAC 0023).

##### Commentary.

*Ophiocordyceps rubroflava* is characterized by bright orange to yellow-orange, cylindrical, soft, fleshy stromata with a distinctive bicolored appearance (red to orange-red fertile part vs. bright orange or white stipe), completely immersed ovoid to oblong-ovate perithecia (275–380 × 105–180 µm), and cylindrical part-spores 3.0–6.0 × 1.0–2.0 µm. It occurs on coleopteran larvae buried in soil in Yunnan Province, China.

Phylogenetically, *O. rubroflava* is strongly supported and clusters with *O. formosana*, *O. jinguangensis*, *O. melolonthae*, and *O. neovolkiana*, but forms a distinct clade (Fig. 1). Morphologically, it differs from *O. formosana* by stromata arising from the host’s back (vs head and tail), a clavate to spherical, red to orange-red fertile head (vs. orange, mostly elliptic), smaller perithecia (275–380 × 105–180 µm vs 453–546 × 265–298 µm), shorter and narrower asci (160–195 × 4.5–6.5 µm vs 366–498 × 8–11 µm), and slightly longer part-spores (3.0–6.0 × 1.0–2.0 µm vs 2–6 × 1–3 µm) (Li et al. 2005). *Ophiocordyceps melolonthae* (Mains 1958) differs from *O. rubroflava* in its longer stromata (3–13 cm vs 3–4.5 cm), sulphur-yellow to mikado-orange coloration, Scarabaeidae host, larger perithecia (360–480 × 170–264 µm vs 275–380 × 105–180 µm), longer asci (210–300 × 6–8 µm vs 160–195 × 4.5–6.5 µm), and longer, narrower part-spores (4–8 × 1–1.5 µm vs 3.0–6.0 × 1.0–2.0 µm). *Ophiocordyceps neovolkiana* differs from *O. rubroflava* in its ovoid to tuberiform fertile part, larger perithecia (340–460 × 140–165 µm vs 275–380 × 105–180 µm), and larger asci (230–300 × 9–10 µm vs 160–195 × 4.5–6.5 µm) (Kobayasi 1941). For differences between *O. rubroflava* and *O. jinguangensis* see the commentary on *O. jinguangensis*.

Table 2 provides a morphological comparison of representative species within the *Ophiocordyceps ravenelii* complex.

**Table 2.** Morphological comparisons of representative species within *Ophiocordyceps ravenelii* complex.

Species	Host/substratum	Stromata/Synnemata (mm)	Perithecia (µm)	Asci (µm)	Ascospores (µm)	Part-spores (µm)	Conidiogenous cells (µm)	Conidia (µm)	References
*O. barnesii*	Coleoptera	Solitary and simple	Immersed, obclavate, 340–430 × 120–230	Cylindrical 150–185 (–220) × 5–10	110–165	32–45 × 2–2.5	–	–	Luangsa-Ard et al. 2010
*O. clavata*	Tenebrionidae larva	Multiple, clavate, pale yellow, long 2–10, 0.5–1.5 wide	Immersed, broad ovoid, 400–500 × 250–350	Cylindrical, 200–300 × 6–8	Cylindrical, long 200–250	5–10 × 1.5–3	–	–	Mongkolsamrit et al. 2024
*O. formosana*	Tenebrionoidea larva (Coleoptera)	Several, yellow to orange, long 14, wide 2–5	Semi-immersed, 453–546 × 265–298	366–498 × 8–11	Cylindrical	2–6 × 1–3	–	–	Li et al. 2005; Li et al. 2016
*O. highlandensis*	Coleoptera larva	Solitary and simple, 3.5–8 cm in height	Immersed, vase-form, ovoid to oblong, 230–410 × 120–200	Narrowly clavate to nearly cylindrical, 140–170 × 5–6.5	Filiform, 130–150 × 1.5–2	(20)33–55 × 1.5–2	–	–	Yang et al. 2015
*O. jinguangensis*	**Elateroidea larva (Coleoptera)**	**Solitary or in pairs, cylindrical, yellow to yellowish brown, long 12–25 mm**	**Immersed, ovoid to oblong-ovate, 283–550 × 175–375**	**Filiform, 235–355 × 6.5–7.5**	**Filiform, 210–305 × 1.5–2.0**	**Cylindrical, 4.5–8.0 × 1.0–2.5**	–	–	**This study**
*O. jinguangensis*	Tenebrionoidea larva (Coleoptera)	Solitary, yellow to brown, 10.2–16.5 × 1–2.2 mm.	Immersed, ovoid to oblong-ovate, 306–496 × 134–223	153.6–273.2 × 5.2–11.8	Filiform, 133.4–187.6 × 1.4–2.4	Cylindrical, 6.7–9.6 × 1.6–2.7	–	–	Zhang et al. 2026
*O. krachonicola*	Nymph of Gryllotalpa orientalis (Gryllidae)	Solitary, cylindrical, dark gray, 37–40 × 1–1.5 mm	Completely immersed, ovoid to obclavate, 460–580 ×	Cylindrical, 250–400 × 4–5	Cylindrical 4–10 × 1	–	–	–	Thanakitpipattana et al. 2020
*O. kuchinaraiensis*	Coleoptera larva	Single, or double, brown, cylindrical, dark brown to black, slightly curved, 5.5–10 cm long, 2–3 mm wide	Semi-immersed, obclavate, 630–820 × 210–300	Cylindrical, 305–525 × 4.5–5.5	Filliform, 430–615 × 1–2	5–20 × 1–2	–	–	Crous et al. 2023
*O. melolonthae*	Scarabaeid larva (Coleoptera)	Clavate, sulphur-yellow to mikado-orange, yellowish brown, 3–13 cm long, 5–15 mm wide	Immersed, ovoid, 360–480 × 170–264	Cylindrical, 210–300 × 6–8	Filiform	4–8 × 1–1.5	–	–	Mains 1958
*O. neovolkiana*	Scarabaeidae larva (Coleoptera).	Solitary or in pairs, 13-18 mm high, fleshy	Immersed, ovoid or ellipsoid, 340–460 × 140–165	Attenuated downwards, 230–300 × 9–10	–	Cylindrical, 2.8–8 × 1.7–2	–	–	Kobayasi 1941
*O. nigrella*	Lepidopteran larva	4.5–10 cm	Superficia, obpyriform, 200–300 × 130–150	Cylindrical	–	26.9–30 × 1–2.9	–	–	He 2019
*O. pseudovariabilis*	Lycidae larva (Coleoptera)	Solitary, multiple, brownish orange to moderate orange, 4–18 mm long, 0.5–1 mm wide	Pseudo-immersed, pyriform, 380–480 × 220–320	Cylindrical, up to 360 µm long, 5–8 µm wide	Cylindrical	5–10 × 1–1.5	*Hirsutella*-like, monophialidic, polyphialidic, 4–15 × 2–5	Fusoid, 4–8 × 0.5–1 µm	Mongkolsamrit et al. 2024
*O. purpureostromata*	Elateridae larva (Coleoptera)	Cylindrical, purple, 7–23 mm long, 0.6–1 mm wide	Immersed, pyriform, 430–450 × 220–250	150–160 × 10	13–23 × 2.5–3	–	–	–	Kobayasi and Shimizu 1980
*O. rubroflava*	**Coleoptera larva**	**Solitary or in groups of 2–8, bright orange to yellow orange, 3–4.5 cm long, 3–8 mm wide**	**Immersed, ovoid to oblong-ovate, 275–380 × 105–180**	**Filiform, 160–195 × 4.5–6.5**	–	**Cylindrical, 3.0–6.0 × 1.0–2.0**	–	–	**This study**
*O. superficialis*	Coleoptera larva	Light gray, grayish brown or black, 1.5 cm long, 0.3–2.0 mm thick	Ovoid to subgloboid, 320–560 × 250–420	Cylindrical, 170–360 × 6–9	Filiform	14–32 × 1.5–2	–	–	Mains 1958
*O. variabilis*	Xylophagidae (Diptera)	Solitary, several, chrome yellow, 2–24 mm long, 0.2–3 mm wide	Immersed, obpyriform, 350–590 × 200–370	Cylindrical, 210–330 × 6	Cylindrical	Cylindrical, 5–10 × 1.5–3	10–19.2 × 2.5	Subcylindrical, 8–12.4 × 1.9–3.1	Hodge et al. 1998

## Discussion

The present study provides a taxonomic revision of the *Ophiocordyceps ravenelii* complex from China. *Ophiocordyceps rubroflava* is recognized as a new species based on its distinct phylogenetic position and morphological characters, while our specimens CXAC 0024 and CXAC 0025 are identified as *O. jinguangensis*, for which an emended description is provided. Both taxa form well-supported independent lineages within the *O. ravenelii* clade, distinct from *O. formosana*, *O. melolonthae* and *O. neovolkiana* (Fig. 1). Morphological characters including stromatal coloration, fertile part shape, perithecial dimensions, and part-spore size correspond to these phylogenetic divergences, demonstrating consistency between genetic and phenotypic datasets.

### Morphological characters and their taxonomic significance in the *Ophiocordyceps ravenelii* complex

Comparative analysis of the *O. ravenelii* complex (Table 2) reveals that morphological characters exhibit varying levels of taxonomic utility, consistent with broader patterns observed in Ophiocordycipitaceae. Host association serves as a diagnostically valuable trait at species level. While the complex predominantly parasitizes Coleoptera, specificity to particular families (*O. jinguangensis* on Elateroidea and Tenebrionoidea, *O. formosana* on Tenebrionoidea) provides reliable discriminatory power. Exceptions to non-coleopteran hosts (*O. krachonicola* on Orthoptera, *O. variabilis* on Diptera) further underscore host range as a key diagnostic feature (Hodge et al. 1998; Li et al. 2005; Shrestha et al. 2016; Tasanathai et al. 2019; Thanakitpipattana et al. 2020). Stromatal characters offer moderate to high taxonomic value. Stromatal coloration, ranging from yellow (*O. jinguangensis*), orange (*O. rubroflava*), purple (*O. purpureostromata*), to black (*O. kuchinaraiensis*), is particularly useful for species recognition. Stromatal size shows discontinuous variation, with extreme values, diminutive in *O. pseudovariabilis*, elongate in *O. kuchinaraiensis* aiding species delimitation. Fertile part morphology (shape, color) further contributes to interspecific differentiation. Perithecial characters exhibit both conserved and variable features. Immersion status is largely conserved (immersed to semi-immersed) with limited species-level value. An exception occurs in *O. nigrella*, whose superficial perithecia represent a distinctive autapomorphy. Shape and size, though overlapping, provide diagnostic ranges in some taxa, *O. kuchinaraiensis*: 630–820 × 210–300 µm (He 2019; Crous et al. 2023). Ascospore and part-spore characters reveal that considerable variation in part-spore length across species (ranging from 2 to 55 µm) enables reliable species-level discrimination within the *O. ravenelii* complex, despite the shared generic synapomorphy of filiform to cylindrical ascospores disarticulating into part-spores, as exemplified by *O. barnesii* (32–45 µm) and *O. formosana* (2–6 µm) (Luangsa-Ard et al. 2010).

The emended description of *O. jinguangensis* based on our specimens documents its occurrence on Elateroidea larvae, expanding the known host range of the *O. ravenelii* complex, which previously included Scarabaeidae and Tenebrionoidea. This suggests that host family-level specialization may be more prevalent within the complex than previously recognized. Whether this reflects true ecological specificity or geographic sampling bias requires further investigation. The occurrence of *O. rubroflava* on Coleopteran larvae similarly highlights the need for precise host identification in future collections.

Both taxa studied herein were collected in Yunnan, contributing to the growing inventory of *Ophiocordyceps* diversity in southwest China. The close relationship among taxa from China, Japan, India, and Vietnam suggests a regional distribution pattern warranting further phylogeographic study. As a closely related member of this complex, *O. formosana* is pharmacologically notable for its antioxidant and antitumor activities and also alleviates hyperglycemia and depression-like behavior in diabetic mice by improving insulin sensitivity, increasing adiponectin levels, and restoring neurotransmitter (5-HT and dopamine) function in key brain regions (Wang et al. 2015; Huang et al. 2016). While the potential of *O. rubroflava* remains to be explored, its distinct morphology warrants recognition as a new species. From a nomenclatural perspective, although *O. formosana* is phylogenetically coherent (Wang et al. 2015), its current combination is invalid under the Code: the combination *O. formosana* (Kobayasi & Shimizu) Wang et al. (2015) is invalid (nom. inval.) per Index Fungorum, lacking a required identifier (Art. 41.5, F.5.1), with the basionym being *Cordyceps formosana* Kobayasi & Shimizu, (1981). This nomenclatural deficiency is hereby corrected by the formal validation of the combination herein: *Ophiocordyceps formosana* (Kobayasi & Shimizu) Wang, Tsai, Tzean & Shen ex Dong, comb. nov., MycoBank MB 863574.

A limitation of this study is that we were unable to obtain pure cultures despite multiple attempts. Consequently, the asexual morphs of both *O. jinguangensis* and *O. rubroflava* remain unknown. Future efforts employing broader culture conditions may be necessary to induce asexual morph formation and elucidate the complete life cycles of these species.

## Supplementary Material

XML Treatment for Ophiocordyceps jinguangensis

XML Treatment for Ophiocordyceps rubroflava

## Additional information

### Artificial Intelligence (AI) use

The authors accept full responsibility for the content of the manuscript, including the disclosure of any use of AI.

No AI tools were used in the preparation of this manuscript.

### Data availability

All of the data that support the findings of this study are available in the main text.
